# Indoleamine 2,3-Dioxygenase 1: A Promising Therapeutic Target in Malignant Tumor

**DOI:** 10.3389/fimmu.2021.800630

**Published:** 2021-12-23

**Authors:** Xiaotian Song, Qianqian Si, Rui Qi, Weidan Liu, Miao Li, Mengyue Guo, Lin Wei, Zhiyan Yao

**Affiliations:** ^1^ Department of Immunology, Hebei Medical University, Shijiazhuang, China; ^2^ Key Laboratory of Immune Mechanism and Intervention on Serious Disease in Hebei Province, Shijiazhuang, China; ^3^ Department of Clinical Laboratory, The People’s Hospital, Pingxiang County, Xingtai, China

**Keywords:** IDO1, tryptophan, kynurenine, tumor immune escape, immunotherapy

## Abstract

Tumorigenesis is a complex multifactorial and multistep process in which tumors can utilize a diverse repertoire of immunosuppressive mechanisms to evade host immune attacks. The degradation of tryptophan into immunosuppressive kynurenine is considered an important immunosuppressive mechanism in the tumor microenvironment. There are three enzymes, namely, tryptophan 2,3-dioxygenase (TDO), indoleamine 2,3-dioxygenase 1 (IDO1), and indoleamine 2,3-dioxygenase 2 (IDO2), involved in the metabolism of tryptophan. IDO1 has a wider distribution and higher activity in catalyzing tryptophan than the other two; therefore, it has been studied most extensively. IDO1 is a cytosolic monomeric, heme-containing enzyme, which is now considered an authentic immune regulator and represents one of the promising drug targets for tumor immunotherapy. Collectively, this review highlights the regulation of IDO1 gene expression and the ambivalent mechanisms of IDO1 on the antitumoral immune response. Further, new therapeutic targets *via* the regulation of IDO1 are discussed. A comprehensive analysis of the expression and biological function of IDO1 can help us to understand the therapeutic strategies of the inhibitors targeting IDO1 in malignant tumors.

## Introduction

Tryptophan (Trp) depletion and kynurenine (Kyn) production promote immunosuppression in different tumor types (1, 2). Indoleamine 2,3-dioxygenase 1 (IDO1) catalyzes the first and rate-limiting enzyme of the essential amino acid Trp catabolism and degrades Trp along a pathway known as the Kyn pathway. In this cascade of enzymatic reactions, several biologically active metabolites are produced, such as Kyn, an immunosuppressive metabolite. Finally, nicotinamide adenine dinucleotide (NAD+) and adenosine triphosphate (ATP) in this process are produced to fuel cellular metabolism (3, 4). The main theory about the function of IDO1 is that Trp availability is locally reduced while bioactive metabolites such as Kyn are increased, which mediate immune regulation and immune tolerance involved in the pathological mechanisms of tumor immune escape. In recent years, with the deepening research, the IDO1 function is more complex than initially assumed. IDO1 is not only an enzyme but also a mediator of a signaling pathway to sustain the regulatory phenotype of a specific set of immune cells (5), which may be associated with the protein conformations of IDO1 in the cells responding to the distinct context (6). Therefore, a full understanding of the expression of IDO1 and biological function may provide more effective immunotherapeutic approaches for a wide range of malignant tumors. Besides IDO1, it has been shown that the other two types of isoenzymes, tryptophan 2,3-dioxygenase (TDO) and indoleamine 2,3-dioxygenase 2 (IDO2), catalyze the same biochemical reaction. However, TDO and IDO2 show higher tissue specificity and much lower enzyme activity than IDO1 that significantly restrict their function in immune regulation. The main role of TDO is involved in maintaining the homeostasis of Trp level and plays a key modulator in brain disease (4, 7, 8). IDO2 was identified as its high homology with IDO1, but the expression and precise activity of IDO2 have not been well elaborated in human tissue due to lower enzyme activity and complexity of human IDO2 transcription (9). Accordingly, this review mainly describes the immunosuppressive mechanisms of IDO1 in tumors.

## Basic Features of Indoleamine 2,3-Dioxygenase 1

IDO1, also known as IDO in the literatures (5, 10), was first identified in rabbit small intestines in 1967. In 1998, IDO1 was described as a molecule associated with immunosuppression in maternal–fetal tolerance (11). It was not until 2006 that the crystal structure of human IDO1 (hIDO1) was first reported (12). In 2017, Lewis-Ballester et al. reported that the crystal structure of the hIDO1–Trp complex and revealed interaction sites of hIDO1 with Trp substrate (13). The resolution of the crystal structure of hIDO1 has shown that it is folded into two domains, including a catalytic large C-terminal domain and a non-catalytic small N-terminal domain, which was connected by a long loop. IDO1 protein contains 403 amino acids, which are intracellular heme-containing dioxygenases (also known as metalloproteins) and encoded by INDO (human chromosome 8p22). Its catalytic activity requires the prosthetic group heme. Along with inactive heme-Fe^3+^ being reduced into active heme-Fe^2+^, IDO1 catalyzes the oxidative cleavage of Trp to produce the intermediate product *N*-formylkynurenine, which is further hydrolyzed to Kyn. By using Trp depletion and Kyn production, IDO1 is considered as an immunomodulatory enzyme involved in anti-inflammation, tumor immune escape, and immunoregulation to promote maternal tolerance toward the allogeneic fetus, suppressing transplant rejection, regulating autoimmune disorders, and so on. In addition to its enzyme activity, a signaling function has recently been described for the phosphorylated form of immunoreceptor tyrosine-based inhibitory motifs (ITIM1 or ITIM2), located at sites in the small non-catalytic domain and the interconnecting loop of IDO1 protein. Albini et al. confirmed that ITIM-related phosphorylation could upregulate or downregulate IDO1 expression in interleukin-6 (IL-6) or transforming growth factor-β (TGF-β)-dominated environments, which suggest that the ITIMs in IDO1 not only control its own stability but also participate in a self-maintaining immunological modulation (14). Therefore, the appropriate regulation of the phosphorylation of ITIMs of IDO1, leading to either enhancing or terminating the expression of IDO1, may provide some innovative strategies in treating malignant tumors. Recently, a separate study confirmed that IDO1-dependent signaling events would activate class IA phosphoinositide 3-kinases (PI3Ks) to produce immunoregulatory phenotype in plasmacytoid dendritic cells (pDCs), accompanied by IDO1 shifting from the cytosol to early endosomes (15). In conclusion, the available evidences indicate that the IDO1 is not only an enzyme in the Kyn pathway but also a moonlighting protein that mediates non-catalytic functions through different mechanisms (16).

## The Expression and Activity of Indoleamine 2,3-Dioxygenase 1

### The Constitutive/Intrinsic Expression of Indoleamine 2,3-Dioxygenase 1

IDO1 is not or weakly expressed under physiological states. It is constitutively expressed in a restricted set of tissues, including the placenta, the mucosa, and lymphoid organs (https://www.proteinatlas.org/ENSG00000131203-IDO1/tissue). For example, IDO1 is mainly expressed in the endothelial cells of the placenta, epithelial cells of the fallopian tube, interstitial cells of the lymph node, and so on (17). Interestingly, some data confirmed that IDO1 expression was increased in select tissues with age (18).

Although IDO1 expression is often silent in normal tissues, the IDO1 expression/activity has been observed in malignant cells. The loss of Bridging Integrator 1 (BIN1; with the features of immunosuppression) or overexpression cyclooxygenase-2 (COX-2) in malignant cells is usually the reason for high constitutive/intrinsic expression of IDO1. The deletion or downregulation of BIN1 in malignant cells enhances IDO1 expression depending on signal transducer and activator of transcription 1 (STAT1) and nuclear factor-kappa B (NF-κB) (19). On the contrary, high BIN1 expression has a favorable prognosis in cancer (20). The up-expression of COX-2 increases its product prostaglandin E2 (PGE_2_) binding to the EP receptor through the autocrine signaling pathway, which activates IDO1 *via* the protein kinase C (PKC) and PI3K pathways (21). Indeed, genetic studies of IDO1 in the mouse suggested there was genetic overlap between COX-2 and IDO1 (22). Litzenburger et al. suggested that constitutive IDO1 expression in human tumor cells was sustained by an autocrine aryl hydrocarbon receptor (AhR)-IL-6-STAT3 signaling loop (23), although the clinical data revealed that the upregulated expression of IDO1 in various human tumor tissues, such as esophageal cancer, thyroid carcinoma, and leiomyosarcoma, was considered to be a worse prognostic factor and a more aggressive tumor phenotype (24–26). However, there was still controversy about the relationship between high expression of IDO1 in tumor-draining lymph nodes (TDLN) and poor clinical outcomes (17, 27).

In summary, there may be some discrepancies in IDO1 expression profiles in different tumor types. Nonetheless, constitutive IDO1 expression in tumor cells is still a key factor to mediate immune evasion, and thus exploring the mechanism of up-expression may guide and pre-evaluate the efficacy of therapeutic approaches by targeting IDO1.

### The Induced/Extrinsic Expression of Indoleamine 2,3-Dioxygenase 1

As noted above, the IDO1 expression is constitutive in some tumor cells. However, it could be also induced to express in tumor cells and intratumoral cells, including DCs, macrophage, endothelial cells, cancer-associated fibroblasts (CAFs), and mesenchymal stem cells (MSCs) (28–34), by a variety of inflammatory stimuli, such as interferon-γ (IFN-γ), tumor necrosis factor-α (TNF-α), IL-32, and IL-6 (33, 35–37). Among the multiple mediators of IDO1 induction, IFN-γ is considered the main inducer of IDO1. Interestingly, tumor-infiltrating lymphocytes (TILs) of the tumor microenvironment (TME) represent the major source of IFN-γ secretion (38–40). IFN-γ inducing IDO1 expression has been extensively studied. For instance, IFN-γ mediates STAT1 to form a homodimer and then binds to the gamma activation sequence (GAS) in IDO1 gene. Meanwhile, IFN-γ also mediates NF-κB and STAT-1-dependent synthesis of IFN-γ-regulated factor 1 (IRF1), which binds to the IFN-stimulated elements (ISREs) in IDO1 gene promoter to induce the transcription of IDO1 (41).

In addition to IFN-γ, there are other cytokines involved in the induction of IDO1. Multiple myeloma cell-derived IL-32γ significantly induced the production of the IDO1 in macrophages through proteinase 3 (PR3) and the downstream STAT3 and NF-κB pathways (33). However, the role of IL-6 in inducing the expression of IDO1 is controversial. It was reported that constitutive IDO1 expression in SKOV-3 and NSCLC human cancer cell lines was sustained by autocrine IL-6 (23). Hepatic CAF-derived IL-6 also differentiated DCs into a regulatory subtype through STAT3 activation (42). In contrast, IL-6 induced IDO1 proteasomal degradation by selectively inducing the interaction between SOCS3 and ITIM of IDO1 in DCs (14, 43). The conflicting results of the IL-6 effect on IDO1 expression suggest that there are different signals in different cells or the complicated environment involved in its expression, which need to be well illustrated in the future.

So far, there are other factors and signaling events involved in IDO1 expression/activity in DCs that have been extensively analyzed. It was reported that tumor cells promote tolerization of DCs through paracrine Wnt5a-mediated signaling. Melanoma-derived Wnt5a promotes the transcriptional expression of IDO1 in nearby DCs by Wnt5a-β-catenin signaling and activates peroxisome proliferator-activated receptor-γ (PPAR-γ) signaling pathway, culminating in enhanced IDO1 activity to establish an immunosuppressive microenvironment (44). Cytotoxic T lymphocyte-associated protein-4-immunoglobulin (CTLA-4-Ig) interacts with B7 molecules as receptors to induce IDO1 expression in DCs (45). TGF-β could trigger immunoregulatory signaling in IDO1, which did not require the catalyst function of IDO1 to induce pDCs for long-term tolerance (5, 14). Interestingly, spermidine, a main arginase 1 (Arg1) product, is required for IDO1 expression and activity by TGF-β in DCs (46).

In addition to the inducers described above, type I IFNs (IFN-α and IFN-β), IL-10, soluble CD83 (sCD83), and toll-like receptor (TLR) ligands such as bacterial lipopolysaccharides (LPSs) are still involved in the modulation of IDO1 expression/activity (47–52). When mitochondrial Lon is overexpressed in oral cancer cells OEC-M1, mitochondrial DNA (mtDNA) is damaged, and then oxidized mtDNA is released into the cytosol to induce IFN-β signaling *via* cytosolic DNA sensors, which upregulates the programmed death ligand-1 (PD-L1) and IDO1 expression (50). Aside from all this, miRNAs are also involved in the regulation of IDO1 expression. *In vitro*, cervical cancer cells secreted exosomal miR-142-5p, which induces IDO1 expression *via* targeting lymphatic AT-rich interactive domain-containing protein 2 (ARID2) to enhance IFN-γ transcription by suppressing promoter methylation (53). On the contrary, miR-153 expression in bladder cancer cells could exert antitumor activity by targeting IDO1 3′-UTR and inhibiting cancer cell Trp metabolism subsequently (54).

Collectively, a great variety of stimuli can affect either directly or indirectly IDO1 expression and activity in different cell types in TME. However, the proportion of these cell types may differ in different tumors and tissues, the exact mechanisms for the distinct expression patterns of IDO1 are only partially revealed, and the functions of overexpressed IDO1 in these cell types are far from completely understood. It is notable that the complex interaction between tumors cells and other cells, especially immunity cells in TME, contributes substantially to exploring the strong IDO1 expression and its particular function.

### Indoleamine 2,3-Dioxygenase 1 and Tryptophan Metabolism

Trp is one of the eight essential amino acids that cannot be synthesized in the human body. In addition to being a building block for proteins synthesis, Trp undergoes complex metabolic pathways, resulting in the production of many active compounds. Less than 2% of Trp is hydroxylated to produce 5-hydroxytryptophan, which is then decarboxylated by an aromatic amino acid decarboxylase to produce 5-hydroxytryptamine (5-HT), an essential neurotransmitter. A very small percentage of Trp can be decarboxylated to produce tryptamine to control the balance between excitatory and inhibitory functions of 5-HT. About more than 95% of the Trp is catalyzed by IDO1 or the other two isoenzymes (IDO2 or TDO), which catalyzes the Trp *via* the Kyn pathway to produce Kyn (55). Kyn is a key component in the synthesis of a number of metabolites, which could convert into 3-hydroxykynurenine (3-HK), 3-hydroxyanthranilic acid (3-HAA), and quinolinic acid. Quinolinic acid finally undergoes a series of chemical reactions to produce NAD+, an important cofactor for redox reactions in mitochondria, while excess carbon skeletons from the Kyn pathway eventually participate in the citric acid cycle to produce ATP. The depletion of Trp and production of Kyn through the Kyn pathway affect the immune cell metabolism and tumor characteristics. It has been confirmed that the IDO1–Kyn–ligand-activated transcription factor (AhR) pathway in thyroid cancer cells would facilitate epithelial-to-mesenchymal transition (EMT) (56), while Kyn depletion *in vivo* would reverse IDO1-mediated cancer immune suppression in an animal model (57).

Although IDO1, IDO2, and TDO may catalyze the same biochemical reactions in the metabolism of Trp, they have different tissue distribution and physiological functions. Unlike IDO1, TDO is mainly found in the liver and neuronal cells and is regulated by glucocorticoid hormones and Trp levels. The main role of TDO is to maintain homeostasis of dietary Trp levels, and there is also evidence that TDO plays a role in immune-related diseases and central nervous system disorders (58, 59). Nevertheless, the recent studies revealed that TDO could be involved in modulating antitumor immune responses and the antitumor immunotherapy efficacy (60, 61), but it did not colocalize with IDO1, at least, in human glioblastoma (62). In most cancers, such as glioblastomas, melanomas, colon carcinomas, lung carcinomas, and endometrium carcinomas, TDO could be detected in pericytes that belonged to morphologically abnormal vessels in the intratumoral rather than tumor cells themselves (62), although the mechanism that triggers TDO expression in tumor pericytes and the relationship between TDO-expression pericytes and abnormal vessels are all unclear, which suggests that TDO may play a proangiogenic role depending on its expression site in certain cancer types. IDO2 is directly adjacent to IDO1 on the same chromosome, is more narrowly expressed, and has much less catalytic efficiency for Trp than IDO1 (63). Although IDO2 was also detected at high levels in some human tumors, the function of IDO2 in tumors is still far from being understood (64, 65). The available evidences support that IDO1, TDO, and IDO2 may be all involved in malignant tumor, but the three differ in the expression, regulatory mechanism, and the role in different TME.

## Indoleamine 2,3-Dioxygenase 1 in Modulating the Immunosuppressive Tumor Microenvironment

**Figure 1:**
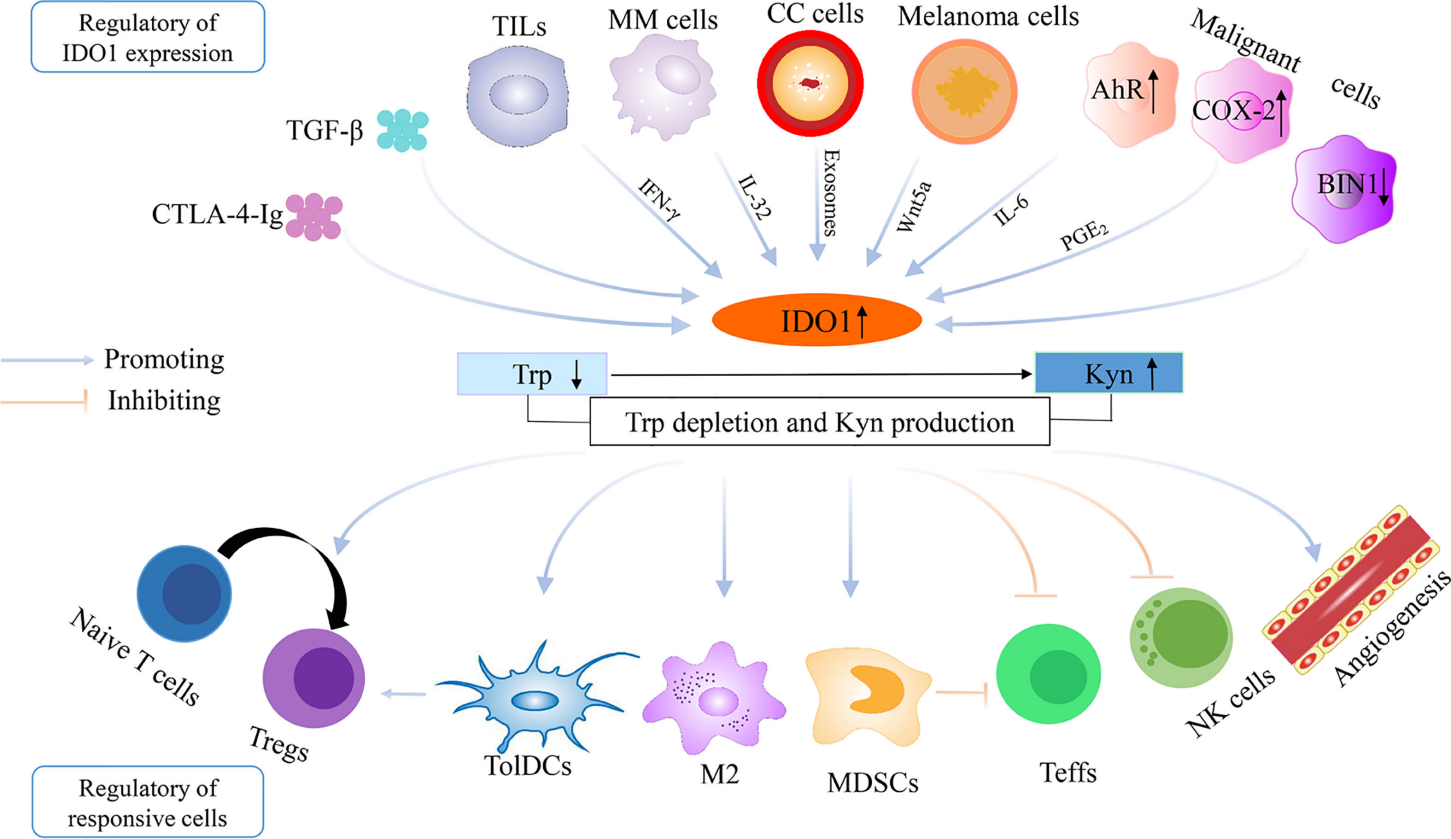
The regulation of IDO1 overexpression and the establishment of immune escape in the tumor microenvironment. IDO1, indoleamine 2,3-dioxygenase 1; Trp, tryptophan; Kyn, kynurenine; TGF-β, transforming growth factor-β; CTLA-4-Ig, cytotoxic T lymphocyte-associated protein-4-immunoglobulin; TILs, tumor-infiltrating lymphocytes; COX-2, cyclooxygenase-2; BIN1, Bridging Integrator 1; AhR, aryl hydrocarbon receptor; IL-6, interleukin-6; CC cells, cervical cancer cells; MM cells, multiple myeloma cells; Teffs, effector T cells; Tregs, regulatory T cells; TolDCs, tolerogenic dendritic cells; MDSCs, myeloid-derived suppressor cells; NK cells, natural killer cells; M2, M2 macrophage.

Initially, the function of IDO1 was described as an innate mechanism of defense against microbial invasion (66, 67), because IDO1 could induce depletion of Trp, an essential amino acid for microbial and parasite proliferation (66–68). In 1998, Munn et al. performed a pioneering experiment showing that elevated IDO (namely, IDO1) expression at the maternal–fetal interface was crucial to prevent immune rejection of fetal allografts (11). Subsequently, extensive studies have demonstrated the immunological regulation role for IDO1 in physiological and pathological states including pregnancy, obesity, transplantation, infectious diseases, autoimmune diseases, neurological diseases, and neoplastic diseases (69–72). In clinical researches, the expression of IDO1 has been found in various tumors such as breast cancer, melanoma, and bladder cancer, which inactivates surrounding immune cells in TME primarily through abnormalities of Trp metabolism (54, 73, 74). Here, the mechanisms reported in the literatures are summarized about IDO1 in the establishment of tumor immune escape (**Figure 1**).

### Dysfunction and Apoptosis of Effector T Cells and Differentiation and Activation of Regulatory T Cells

The effect of IDO1 on T cells is based on the Trp “starvation” theory. 1) T cells are especially sensitive to low Trp concentrations, which arrest T cells in the mid-G1 phase of the cell progression cycle (75). 2) The Trp depletion can inhibit T-cell proliferation through the activation of kinase general control non-derepressible 2 (GCN2), a molecular sensor of Trp deprivation, and its downstream phosphorylated eukaryotic initiation factor 2 (eIFα) (76). Furthermore, activation of GCN2 also promotes Treg differentiation, enhances Treg activity, and collaborates with phosphatase and tensin homolog (PTEN) signaling to maintain the suppressive phenotype of Tregs (77). Paradoxically, GCN2 does not mediate suppression of antitumor T-cell responses by Trp catabolism in experimental melanomas (78), and GCN2 is required for normal cytotoxic T-cell function (79), which suggests that the immune regulatory role of GCN2 in subsets of T cells may depend on the complex context in different types of tumors. 3) The Trp shortage can also inhibit the mTOR signaling pathway, which leads to impairment of T-cell function (80). In addition to the depletion of Trp, accumulation of Trp catabolite, including Kyn and downstream derivative metabolites, would also inhibit T effector cell activation and induce Treg differentiation. For instance, Kyn could promote AhR nuclear translocation and then increase the transcription of Foxp3, a marker of Tregs (81). And the activation in Tregs could modulate M2-like macrophage activity, which contributes to the establishment of a myeloid-enriched immunosuppressive TME (82).

### Tolerance of Dendritic Cells and Myeloid-Derived Suppressor Cells and Suppression of Natural Killer Cells Proliferation and Functions

In addition to suppressing the immune effects of T cells, it is generally considered that IDO1 also exerts immunosuppressive effects by regulating the function of innate immune cells, such as DCs, myeloid-derived suppressor cells (MDSCs), and natural killer (NK) cells. IDO1 normally has low basal expression in DCs but is rapidly induced such as by IFN-γ in inflamed tissues, especially in mature, immunogenic myeloid DCs, which are involved in the regulation of immune homeostasis (83, 84). However, in the tumor region, there is a set of DCs highly expressed IDO1 with a high capacity to support immune tolerance. Especially, under the presence of TGF-β in TME, the ITIM1 motif of IDO1 is phosphorylated, which reprograms DCs to the immune tolerance phenotype and leads to sustained IDO1 expression through a positive feedback loop (14). And IDO1-expressing DCs also induce Treg proliferation (85). MDSCs are composed of multiple myeloid cells that are arrested at different stages of lineage development, which would be recruited to the TME by IDO1 overexpressing tumor cells, and then MDSCs inhibit T-cell function and reduce tumor response to immunotherapy in an IDO1-dependent manner (86–89).

NK cells are known as one of the most important innate immune cells with potent antitumor activity. In TME, however, tumor cells would suppress NK cell cytotoxicity and inhibit the expression of activating receptors on the surface of NK cells, such as NKG2D and NKp46, by IDO-induced Kyn production (90). But against this, Nafia et al. demonstrated that the proliferation and granzyme B production of NK cells were inhibited by GDC-0919 (an innovative IDO1 inhibitor) through upregulation of inhba (encoding for the inhibin—a member of the Tgfbeta signaling) (91). Certain IDO1 inhibitors unexpectedly impair NK cell-mediated killing in tumors, which suggests that we consider the inhibitory mechanisms of different IDO1 inhibitors and their effects on others cells, especially immune cells.

### Neovascularization of Tumor

Tumor growth depends on continuous and extensive angiogenesis, which is a major pathway for tumor metastasis. Among them, vascular endothelial growth factor (VEGF) plays an important role in tumor angiogenesis. It has been found that IDO1 could increase angiogenesis through IL-6/STAT3/VEGF signaling (54). The expression of IDO1 in MDSCs has been implicated in promoting neovascularization through GCN2, which shifts the balance between the inflammatory cytokines IFN-γ and IL-6 (92). *In vivo* experiments also showed that the blood vessel density in the tumor was significantly reduced, and the tumor growth and metastases were impeded in IDO1-deficient mice (93, 94).

Furthermore, IDO1 can be induced in endothelial cells, CAFs, and MSCs, which could participate in mediating an immunosuppressive TME, for instance, supporting cancer cells to evade tumor dormancy (95), impairing NK cell function (96), and inducing Treg expansion (28). However, accumulating evidences about the mechanism of IDO1 action in immunosuppression indicate that not all immunosuppressive effects of IDO1 can be explained through the Trp depletion/Kyn accumulation theory (97). Besides, contrary to what is generally hypothesized in suppressing the immune effects of T cells, IDO1 would supply the required energy for T-cell survival and proliferation by increasing free fatty acid oxidation (98).

In conclusion, IDO1 is a key mediator in the establishment of tumor immune escape. Nonetheless, a greater understanding is needed about the exact mechanisms in the immunosuppressive effects of Trp catabolism by IDO1 derived from different cells in the different TME. Besides, detailed information about the differences related to the catalytic and non-catalytic functions of IDO1 is needed to elucidate this.

## The Significance of Targeting Indoleamine 2,3-Dioxygenase 1 in Tumor Therapy

Recently, wide use of immune checkpoint inhibitors (ICIs), which mainly target CTLA-4 and the programmed death receptor/ligand 1 (PD-1/PD-L1) in cancer immunotherapies, improved durable responses in some advanced cancer patients (99). Nevertheless, these existing checkpoint inhibitors have shown substantial benefit to only some of the patients, while the majority of patients do not respond to this approach, and even treatment-induced resistance would arise in the initial treatment responders, and life-threatening adverse effects would occur after ICI treatment (100–102). Therefore, it is very important to develop a reasonable immunotherapy strategy targeting different immunosuppressive points in TME. Several studies suggest there may be the non-T-cell-inflamed TME (so-called cold tumors) where checkpoint inhibitors are not effective in this group of patients (103). Brown et al. provided evidence of adaptive resistance to anti-CTLA-4 treatment due to upregulation of IDO1 in HCC (104). That is, ICIs in combination with inhibiting IDO1 may improve therapeutic benefit in tumors overexpressed IDO1, which could also drive inflammation in the TME and transform “cold” tumors to “hot” tumors.

So far, there are many small molecule compounds such as IDO1 inhibitors that have been reported to treat cancers alone or in combination with ICIs. 1-Methyl-d-tryptophan (d-1-MT), considered as a first-generation IDO1 enzyme inhibitor, is an analog of Trp. In addition to terminating immune tolerance, d-1-MT can also block the dormancy of tumor-repopulating cells (TRCs) and induce apoptosis through the IDO1-blocking/P53/reactive oxygen species (ROS)-mediated pathway (105). In a phase II trial, the combination of indoximod (d-1-MT) and pembrolizumab (PD-1 checkpoint inhibitor) showed encouraging safety and efficacy in patients with advanced melanoma (106). Controversially, in another independent phase III trial, the efficacy of epacadostat (direct inhibitor of IDO1 enzyme activity) in combination with pembrolizumab was not superior to pembrolizumab monotherapy in patients with unresectable or metastatic melanoma (107). In addition, a series of studies by Zhai et al. showed that in tumor cells, IDO1 suppressed the antitumor immune response by increasing the expression of complement factor H (CFH) and factor H-like protein 1 (FHL-1) instead of its association with Trp metabolism in human glioblastoma, and there was a survival advantage mediated by ICIs requiring non-tumor cell IDO1 enzyme activity in mouse glioblastoma. Oppositely, the combination of radiation and PD-1 antibody treatment efficacy required to inhibit IDO1 enzyme activity in non-tumor cells from another study of mouse glioblastoma model (97, 108, 109). The reason for the controversial conclusion may be that the immunosuppressive effects of IDO1 in the organism are not isolated, and there are multiple factors involved, such as the differentiation degree, the invasion degree, lymph node metastasis, clinical stage of the tumor, the different combinations of inhibitors, the infiltration of T effector cells in the tumor lesion, the host cell IDO1 origin, the enzyme activity versus non-enzyme effects of IDO1 in tumor lesion or TDLN, and age of the subject, all of which need to be considered comprehensively in order to better apply and develop IDO1-targeted drug and new combined therapeutic strategies in the clinical setting.

In addition to targeting IDO1 inhibiting, blocking the AhR pathway would overcome the limitation of single IDO1 targeting agents, particularly in combination with ICIs (82). Therefore, the targeted blockade of IDO1 or IDO1-driven metabolism pathway represents a promising therapeutic pathway. Meanwhile, IDO1 inhibitors combined with other therapies should be considered as an effective strategy in tumor immunotherapy, such as effectively suppressing tumor growth by synergizing photothermal therapy (PTT), radiotherapy, or chemotherapy (110–112). With the discovery of cancer tissue expression IDO1 or TDO or both, IDO1/TDO combined inhibitors have become a study focus (113–115). However, at odds with IDO1 inhibitors, TDO inhibitors are effective in synergistic immunotherapy with ICIs even though there is little or no TDO expression in cancers, which may be because the inhibitor of TDO could block hepatic TDO to increase systemic Trp levels (60, 113). More surprisingly, in viral hepatitis, the inhibition of TDO or IDO (both IDO1 and IDO2) separately leads to dichotomous outcomes. TDO could participate in the Kyn pathway as IDO1 does, but both also differ in mediating inflammation (116, 117). In regard to TDO in the tumor, although the mechanisms to regulate the TDO expression and its separate role in maintaining Trp homeostasis are all unclear as yet, TDO could be regarded as a candidate after resistance to IDO1 inhibitors, as well as the circulating level of Trp may be an indicator to evaluate the efficacy of inhibitors of TDO in tumor immunotherapy. Anyway, one thing should be confirmed that the mechanism of their expression and activation in the different cell types needs to be understood first, which could guide the development and applications of IDO1 inhibitors and IDO1/TDO combined inhibitors.

Due to the short half-life of small molecule inhibitors, the lack of patient stratification based on IDO1 expression, the option of combination with therapy ICIs, and inhibitors targeting IDO1 have so far failed to show therapeutic benefit in the animal model research or even in clinical trials (118–120). For instance, IDO1 inhibitor combination with PD-L1 blockade did not cause a synergistic effect in sarcoma (91). Therefore, more and more new strategies of inhibiting the expression of IDO1 have been explored. Phan et al. found that attenuated *Salmonella* typhimurium (ST) delivering an shRNA plasmid targeting IDO1 can reduce intratumoral IDO1 levels more effectively than epacadostat (121), while locked nucleic acid (LNA)-modified antisense oligonucleotides (ASOs) could inhibit IDO1 expression in cancer cells, exhibiting longer exposure times and more engaged targets than epacadostat (122). Besides, there may be other Trp metabolizing enzymes involved in tumor immune escape, such as interleukin-4-induced-1 (IL4i1), but at this point, the biology and expression of IL4i1 are still poorly understood (123).

In addition to being a target of antitumor therapy, targeted IDO1 can be considered as an independent prognostic value and predictive biomarker. High proportions of PD-L1+ and IDO1+ TAMs are associated with unfavorable outcomes in classical Hodgkin’s lymphoma patients treated with standard chemotherapy (34). Moreover, there is clinical evidence that IDO1 gene expression in the urine of men indicates a high risk of prostate cancer development (36, 124). And in non-small cell lung cancer, the high serum Kyn/Trp levels are also associated with early progression and a low prognosis (125). Even though, as mentioned above, the up-expression IDO1 has been described in various human tumor tissues not only in tumor cells but also in other components of the TME, and the IDO1 expression status in patients has also been explored in some clinical trials to assess its relevance with poor prognosis (126), not all tumor progression or poor prognosis has a positive correlation with high IDO1 expression (127, 128). Also, the current clinical trial data of IDO1 activity assessment are mainly derived from serum Kyn or Trp levels. In fact, the consumption of Trp and the accumulation of Kyn do not always happen simultaneously in human cancers, and the immunosuppression effects of IDO1 in TME do not just depend on its enzyme activity. On the contrary, its enzyme activity may also contribute to the response to ICI therapy (97, 108, 129, 130). Therefore, the high IDO1 expression is not a single indicator to decide whether to choose IDO1 inhibitors, and the IDO1 activity assessment may also need multiple factors, including the concentrations of Trp and Kyn as well as the Kyn/Trp ratio in human cancers.

## Conclusion

Overall, the important role of IDO1 in tumoral immune escape renders the IDO1 pathway a potential target for adjuvant treatment. IDO1 inhibitors are widely studied in various cancers as monotherapy or in combination with other therapies in preclinical and clinical trials. It is remarkable, however, that the complex mechanism of regulating IDO1 expression and its different biological effects depending on the context or cell types may render its clinical development complicated. So more researches are needed to elucidate the mechanisms of immunotherapy against IDO1 and how IDO1 works in combination therapy. And further understanding of the immunobiological properties of IDO1, individual IDO1 expression levels, the optimal drugs targeting IDO1, and combination therapy strategies would lead to favorable treatment for patients with malignant tumors. Besides, it is important to explore the exact role of other Trp metabolizing enzymes, Kyn, and its downstream metabolites in tumoral immune escape.

## Author Contributions

XS, QS, and ZY edited the manuscript. ZY revised the whole manuscript about important intellectual content. XS, QS, ZY, RQ, WL, ML, MG, and LW wrote parts of the manuscript. All authors contributed to the article and approved the submitted version.

## Funding

This work was supported by grants from the National Science Foundation of China (81702827, 81801560), the Science and Technology Planning Project of Hebei Province (H2019206614), and the Science and Technology Research Projects of the Colleges and Universities of Hebei Province (ZD2021071).

## Conflict of Interest

The authors declare that the research was conducted in the absence of any commercial or financial relationships that could be construed as a potential conflict of interest.

## Publisher’s Note

All claims expressed in this article are solely those of the authors and do not necessarily represent those of their affiliated organizations, or those of the publisher, the editors and the reviewers. Any product that may be evaluated in this article, or claim that may be made by its manufacturer, is not guaranteed or endorsed by the publisher.
